# Enhancing the energy spectrum of graphene quantum dot with external magnetic and Aharonov-Bohm flux fields

**DOI:** 10.1016/j.heliyon.2019.e02224

**Published:** 2019-08-06

**Authors:** Fernando Adan Serrano Orozco, Juan Gerardo Avalos Ochoa, Xochitl Cabrera Rivas, Jose Luis Cuevas Figueroa, Hugo Moises Martinez Carrada

**Affiliations:** a Instituto Politécnico Nacional, Escuela Superior de Ingeniería Mecanica y Eléctrica Unidad Culhuacan, CDMX 04430, Mexico; b Yachay Tech Univerity, School of Physical Sciences and Nanotechnology, 100115, Urcuqui, Ecuador

**Keywords:** Quantum mechanics, Dirac-Weyl equation, Energy eigenvalues, Graphene quantum dot, AB-flux field, Landau Levels

## Abstract

In this paper, we have to apply the Dirac-Weyl equation to find the analytical energy eigenvalues of the graphene quantum dot interacting in the presence of AB-flux field and external magnetic field. We find that the energy eigenvalue of the graphene quantum dot decreases with both magnetic and AB-flux field but the effect of AB-flux field is more dominant. By ameliorating the intensity of the AB-flux field and keeping the magnetic field constant, the quantum-dot states entangled to produce Landau Levels. We show that besides using the graphene sheet and external magnetic field, the Aharonov-Bohm AB-flux field could as well be used to manipulate the carriers state energies in graphene.

## 1 Introduction

Graphene can be described as a single layer of carbon atoms that are integrated together in a repeating pattern of hexagons. It is the thinnest substance ever made. It is the basic structural element of other allotrope, including charcoal, graphite and fullerenes carbon nanotubes. One could also consider it as an indefinitely large aromatic molecule. It has exceptional strength, thermal conductivity, and electric conductivity. The potential applications of graphene is not limited to faster computer chips, it also important in designing hyper-efficient solar cells, flexible touchscreens and desalination membranes [1]. It has also shown a promising future for nanoelectronics material. An important class of graphene nanomaterials with exceptional luminescence properties is graphene quantum dot.

Graphene quantum dots are kind of 0D material with characteristics derived from both graphene and carbon dots [2]. It is becoming an advanced multifunctional material because of its unique optical, electronic, spin and photoelectric properties induced by the quantum confinement and edge effects. Graphene quantum dots are not single-layer graphene domains, but multi-layer formations containing up to 10 layers of reduced graphene oxide ranging from 10nm to 60nm in size.

Manufacturing graphene-based quantum structures seems to be of great challenge for its potential application in electronic devices because of the Klein tunneling effect [3] which hinders the confinement of the carriers. To overcome this difficulty, the carriers states energies in graphene can be manipulated either by using infinite graphene sheet or via external magnetic field which leads to the manifestation of Landau levels for an infinite graphene sheet [4].

Magnetic field induced confinement-deconfinement transition in graphene quantum dots was investigated in Ref. [5]. Schnez et al. [6] reported analytical study on the energy spectrum of a graphene quantum dot in a perpendicular magnetic field. The magnetic field dependence of energy levels in gapped single-layer and bilayer graphene quantum dots have been studied analytically in terms of the Dirac equation by Recher et al. [7]. Electronic and optical properties of a circular graphene quantum dot in a magnetic field were studied in Ref. [4].

It was found in Ref. [8] that energy spectrum of graphene with respect to dot size and external magnetic field are quantitatively similar to one another. Energetic model to describe the edge elastic properties of defect-free single-layer graphene sheets was proposed in Ref. [9]. It was found in Ref. [10] that the optical properties of graphene quantum dots are tuned by the size, the type of the edge, and the external magnetic field [10], [11].

Beside the graphene sheet and external magnetic field, we suggest that AB-flux field could also be used to manipulate the carriers state energies in graphene. We refer the readers to Ref. [12] for a more comprehensive study on AB-flux field. Moreover, Ribeir et al. [13] presented a magneto-photoluminescence study of type-II InP/GaAs self-assembled quantum dots, revealing the Aharonov-Bohm-type oscillations for neutral excitons when the hole ground state changes its angular momentum.

Linearity in the behavior of energy spectrum of graphene at the Brillouin zone can be described by the Dirac-Weyl equation which is a characteristic of relativistic massless particles. The study of problems involving Dirac-Weyl Hamiltonian in continuous limit in graphene have been investigated in several literature [14], [15], [16], [17], [18], [19].

Motivated by the considerable interest in studying Dirac-Weyl Hamiltonian in continuous limit in graphene, in this paper, we use the Dirac-Weyl equation to find the energy eigenvalue of a circular graphene quantum dot under the influence of magnetic field and AB-flux field. In section 2, we give a theoretical formulation of the problem. Bound state solution is presented in section 3. Finally, in section 4, we give a brief conclusion.

## 2 Background

In this section, we give theoretical formulation of the problem. In order to achieve our aim of this study, we shall solve the Dirac-Weyl equation in cylindrical coordinate by taking into accounts, the homogeneous magnetic and AB-flux fields perpendicular to the graphene sheet. Thus, the Hamiltonian for this problem becomes

(1) $$H=v(\overset{\to }{p}+e\overset{\to }{A})\cdot \overset{\to }{\sigma }+\tau V(x,y)\sigma_{z},$$

where vector potential $\overset{\to }{A}={\overset{\to }{A}}_{1}+{\overset{\to }{A}}_{2}$ [20] with ${\overset{\to }{A}}_{1}=(Br/2)\overset{ˆ}{\varphi }$, ${\overset{\to }{A}}_{2}=\phi_{AB}/(2\pi r)\overset{ˆ}{\varphi }$. $\overset{\to }{B}=B\overset{ˆ}{z}$, $\overset{\to }{\nabla }\times {\overset{\to }{A}}_{1}=\overset{\to }{B}$. ${\overset{\to }{A}}_{2}$, $\overset{\to }{\nabla }\cdot {\overset{\to }{A}}_{2}=0$. Thus $\overset{\to }{A}=((Br/2)+\phi_{AB}/(2\pi r))\overset{ˆ}{\varphi }$. Fermi velocity is denoted by $v=10^{6}\;\text{m}/\text{s}$ and τ=±1 distinguishes the two valleys $K^{\prime }$ and *K*. $\overset{\to }{\sigma }=(\sigma_{x},\sigma_{y})$ are Pauli's spin matrices which act on sublattice components A and B of the spinor wave function. It is necessary to transform the Hamiltonian to cylindrical coordinates. To do this, firstly, we write $\sigma_{x}$, $\sigma_{y}$ and $\sigma_{z}$ in cylindrical coordinates as:

(2) $$\sigma^{r}=\left(\begin{array}{cc} 0 & e^{-i\varphi }\\ e^{i\varphi } & 0 \end{array}\right),\;\;\sigma^{\varphi }=i\left(\begin{array}{cc} 0 & -e^{-i\varphi }\\ e^{i\varphi } & 0 \end{array}\right)\;\text{and}\;\sigma^{z}=\left(\begin{array}{cc} 1 & 0\\ 0 & -1 \end{array}\right),$$

respectively with ħ=1. Hence, the Hamiltonian becomes

(3) $$H=v\left(-i\frac{\partial }{\partial r}\overset{ˆ}{r}-\frac{i}{r}\frac{\partial }{\partial \varphi }\overset{ˆ}{\varphi }-i\frac{\partial }{\partial r}\overset{ˆ}{z}+\frac{eBr}{2}\overset{ˆ}{\varphi }+\frac{e\phi_{AB}}{2\pi r}\overset{ˆ}{\varphi }\right)\cdot \left(\sigma^{r}\overset{ˆ}{r}+\sigma^{\varphi }\overset{ˆ}{\varphi }\right),$$

and the confinement potential becomes V(x,y)=V(r) with $r=\sqrt{x^{2}+y^{2}}$. To confine the carriers inside quantum dot, we consider the circular well potential which is defined as V(r)=0 for r≤R and V(r)=∞ for r>R. Thus for V(r)=0, we can find the eigenvalue of the problem via HΨ=EΨ, where $\Psi =(\Psi_{1}(r,\varphi ),\Psi_{2}(r,\varphi ))$ is the two-component spinor with $\Psi_{1}(r,\varphi )=e^{im\varphi }\chi_{A}$ and $\Psi_{2}(r,\varphi )=ie^{i(m+1)\varphi }\chi_{B}$. Since the Hamiltonian commutes with the operator of total angular momentum, then the energy eigenspinors take the form $\Psi ={\left[e^{im\varphi }\chi_{A},ie^{i(m+1)\varphi }\chi_{B}\right]}^{T}$, where m=0,±1,±2,... denotes the total angular momentum quantum number. The two components of the wave function $\chi_{A}$ and $\chi_{B}$ correspond to sublattice A and B. Substituting this expression into the Dirac equation, we find the following coupled differential equations:

(4) $$v\frac{d\chi_{B}(r)}{dr}+v\left(\frac{m+1}{r}+\frac{eBr}{2}+\frac{e\phi_{AB}}{2\pi r}\right)\chi_{B}(r)=E\chi_{A},-v\frac{d\chi_{A}(r)}{dr}+v\left(\frac{m}{r}+\frac{eBr}{2}+\frac{e\phi_{AB}}{2\pi r}\right)\chi_{A}(r)=E\chi_{B}.$$

On solving equations (4) simultaneously, we obtain the following Schrödinger-like differential equation satisfying $\chi_{A}$

(5) $$\frac{d^{2}\chi_{A}(r)}{dr^{2}}+\frac{1}{r}\frac{d\chi_{A}(r)}{dr}-\left(\frac{{\left(m+\varsigma \right)}^{2}}{r^{2}}+\frac{m+\varsigma +1}{l_{\beta }^{2}}-k^{2}+\frac{r^{2}}{4l_{\beta }^{4}}\right)\chi_{A}(r)=0,$$

where we have introduced parameters $\varsigma =\phi_{AB}/\phi_{0}$ with $\phi_{0}=hc/e$ for simplicity and $l_{\beta }={\left(eB\right)}^{-1/2}$ denotes the magnetic length. The wave vector *k* is related to the energy via the expression k=E/(vħ). Furthermore, a similar equation satisfying $\chi_{B}$ can be obtained as

(6) $$\frac{d^{2}\chi_{B}(r)}{dr^{2}}+\frac{1}{r}\frac{d\chi_{B}(r)}{dr}-\left(\frac{{\left(m+\varsigma +1\right)}^{2}}{r^{2}}+\frac{m+\varsigma }{l_{\beta }^{2}}-k^{2}+\frac{r^{2}}{4l_{\beta }^{4}}\right)\chi_{B}(r)=0.$$

## 3 Calculation

In this section, we utilize the formula method (FM) [21] to solve equations (5) and (6). One of the calculation tools employed in solving the Schrödinger-like equation including the centrifugal barrier and/or the spin-orbit coupling term is FM. This method was proposed recently in Ref. [21]. For a given potential, the idea is to convert the Schrödinger-like differential equation into the form given by equation (1) of Ref. [21], i.e.

(7) $$\Phi^{\prime \prime }(s)+(k_{1}-k_{2}s)/(s-k_{3}s^{2})\Phi^{\prime }(s)+(P_{1}s^{2}+P_{2}s+P_{3})/(s^{2}{\left(1-k_{3}s\right)}^{2})\Phi (s)=0,$$

via an appropriate coordinate transformation of the form s=s(r). If the problem is exactly solvable, then, the corresponding wave function can be obtained, via

(8) Φ(s)=Nnsk4(1−k3s)k5F12(−n,n+2(k4+k5)+k2/k3−1;2k4+k1,k3s)

and

(9) limk3→0⁡Φ(s)=Nnsk4exp(−k5s)F11(−n,2k4+k1,(2k5+k2)s),withP2−k4k2−nk22k4+k1+2n=k5,

where

(10) $$k_{4}=\frac{(1-k_{1})+\sqrt{{\left(1-k_{1}\right)}^{2}-4P_{3}}}{2}\;\lim_{k_{3}\to 0}k_{4}=\frac{(1-k_{1})+\sqrt{{\left(1-k_{1}\right)}^{2}-4P_{3}}}{2}\;\;\;\text{and}\;k_{5}=\frac{1}{2}+\frac{k_{1}}{2}-\frac{k_{2}}{2k_{3}}+\sqrt{{\left[\frac{1}{2}+\frac{k_{1}}{2}-\frac{k_{2}}{2k_{3}}\right]}^{2}-\left[\frac{P_{1}}{k_{3}^{2}}+\frac{P_{2}}{k_{3}}+P_{3}\right]},\lim_{k_{3}\to 0}k_{5}=-\frac{k_{2}}{2}+\sqrt{{\left(\frac{k_{2}}{2}\right)}^{2}-P_{1}},$$

with $N_{n}$ being the normalization constant. In order to apply this method, we now introduce a new transformation of the form $s=r^{2}$ through which equation (5) becomes

(11) $$\frac{d^{2}\chi_{A}(s)}{ds^{2}}+\frac{1}{s}\frac{d\chi_{A}(s)}{ds}-\frac{1}{s}\left(\frac{{\left(m+\varsigma \right)}^{2}}{s}+\frac{m+\varsigma +1}{l_{\beta }^{2}}-k^{2}+\frac{s}{4l_{\beta }^{4}}\right)\chi_{A}(s)=0.$$

Thus, comparing equation (11) with (7), we found that $k_{1}=1$, $k_{2}=k_{3}=0$, $P_{1}=-1/(16l_{\beta }^{4})$, $P_{2}=k^{2}/4-(m+\varsigma +1)/(4l_{\beta }^{2})$, $P_{3}=-{\left(m+\varsigma \right)}^{2}/4$ and hence, using equation (9), we obtain the solution as

(12) χA(s)=Nmsm+ς2exp⁡(−s4lβ2)F11(m+ς+1−k2lβ22,m+ς+1,s2lβ2)=Cmsm+ς2exp⁡(−s4lβ2)L(k2lβ22−(m+ς+1),m+ς,s2lβ2),

where we have utilized the following relation between Laguerre polynomial and hypergeometric functions, specifically the confluent hypergeometric functions

(13) Lnα(x)=(n+αn)M(−n,α+1,x)=(α+n)nn!F11(−n,α+1,x).

We refer the readers to Ref. [21], [22], [23] for more details and general examples involving the application of formula method. From equation (4), we have

(14) $$\chi_{B}(s)=-\frac{v}{E}\left[\chi_{A}^{\prime }(s)\frac{ds}{dr}-\left(\frac{m+\varsigma }{\sqrt{s}}+\frac{\sqrt{s}}{2l_{\beta }^{2}}\right)\chi_{A}(s)\right]=\frac{i}{kl_{\beta }^{2}}C_{m}s^{\frac{m+\varsigma +1}{2}}\exp\left(-\frac{s}{4l_{\beta }^{2}}\right)[L\left(\frac{k^{2}l_{\beta }^{2}}{2}-(m+\varsigma +1),m+\varsigma ,\frac{s}{2l_{\beta }^{2}}\right)+L\left(\frac{k^{2}l_{\beta }^{2}}{2}-(m+\varsigma +2),m+\varsigma +1,\frac{s}{2l_{\beta }^{2}}\right)].$$

Using equations (12) and (14), $\Psi_{1}(r,\varphi )$ and $\Psi_{2}(r,\varphi )$ can be determined, respectively. Now, we use the boundary condition that the outward current at the graphene edge is zero [24], i.e. $\Psi_{2}(r,\varphi )/\Psi_{1}(r,\varphi )=i\tau e^{i\varphi }$, leads to the energy expression

(15) $$\begin{array}{c} \frac{R}{l_{\beta }}\frac{L\left(\frac{k^{2}l_{\beta }^{2}}{2}-(m+\varsigma +1),m+\varsigma ,\frac{R^{2}}{2l_{\beta }^{2}}\right)+L\left(\frac{k^{2}l_{\beta }^{2}}{2}-(m+\varsigma +2),m+\varsigma +1,\frac{R^{2}}{2l_{\beta }^{2}}\right)}{L\left(\frac{k^{2}l_{\beta }^{2}}{2}-(m+\varsigma +1),m+\varsigma ,\frac{R^{2}}{2l_{\beta }^{2}}\right)} \end{array}=\tau kl_{\beta }.$$

**Figure 1:**
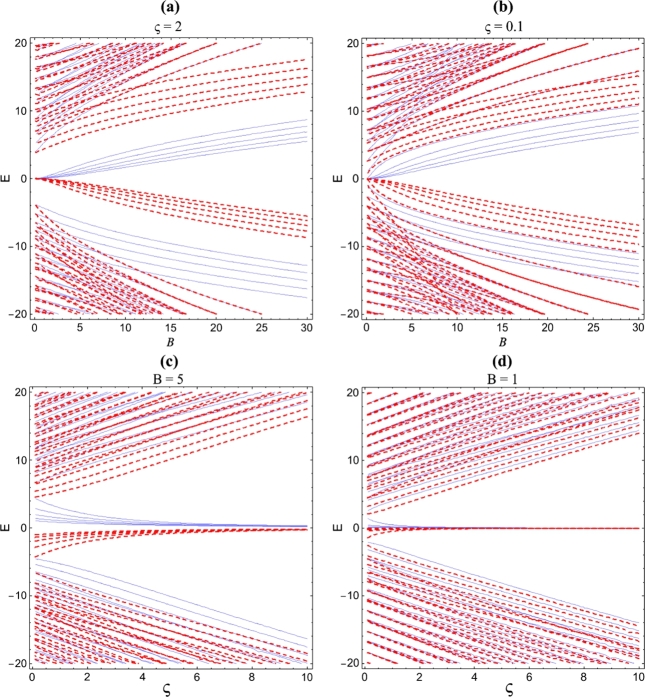
(a) Energy spectrum of graphene quantum dot as a function of magnetic field for *ζ* = 2. The same as (a) but for *ζ* = 2. (c) Energy spectrum of graphene quantum dot as a function of AB-flux field for *B* = 5. The same as (a) but for *B* = 1.

The above expression can be numerically solved for *E* using the standard root finding methods. We have used Wolfram Mathematica 11 for this and the result has been shown in Fig. 1 where we have considered variable magnetic and AB-flux field. We found that there some gaps in the energy which is as a consequence of the infinite mass boundary conditions. The energy spectrum decreases with both magnetic and AB-flux field but the effect of AB-flux field is more dominant. By ameliorating the intensity of the AB-flux field and keeping the external magnetic field constant, the quantum-dot states integrate to produce Landau Levels. Now we proceed to the study of limit $R/l_{\beta }\to \infty$. It should be noted that $R/l_{\beta }$ monitors the transition from a region where the energies of the electrons are dominated by confinement to Landau levels. In this case, it is necessary to obtain the series expansion of Laguerre polynomials in equation (15). We achieve this via:

(16) $$L(n,\alpha ,x)=\frac{(n+a)!}{\alpha !n!}\frac{\Gamma (\alpha +1)}{\Gamma (-n)}e^{x}x^{-(n+\alpha +1)}\left[1+O\left(\frac{1}{\left|x\right|}\right)\right],$$

and consequently, we have

(17) $$\frac{\left(\frac{k^{2}l\beta^{2}}{2}-1\right)!\Gamma (m+\varsigma +1)}{\left(\frac{k^{2}l\beta^{2}}{2}-m-\varsigma -1\right)!\Gamma \left(\frac{k^{2}l\beta^{2}}{2}+m+\varsigma +1\right)}=\frac{\left(\frac{k^{2}l\beta^{2}}{2}-1\right)!\Gamma (m+\varsigma +2)}{(m+\varsigma +1)\left(\frac{k^{2}l\beta^{2}}{2}-m-\varsigma -2\right)!\Gamma \left(-\frac{k^{2}l\beta^{2}}{2}+m+\varsigma +2\right)},$$

which yields analytical expression for the graphene energy in the presence of AB-flux and external magnetic fields as

(18) $$E_{m}=\frac{v}{l_{\beta }}\sqrt{2(m+\varsigma +1)}.$$

When ς=0, equation (18) reduces to Landau levels for graphene.

## 4 Discussion & conclusion

In this paper, we have described a way of confining Dirac-Weyl quasiparticles in graphene. The energy level of the graphene quantum dot has been obtained in the presence of an external magnetic field and AB-flux field. In Fig. 1, we have plotted the energy spectrum as a function of m=−4:1:4 for various magnetic filed intensity and AB-flux field.

The series of crossing and anticrosings that appear in the plot of energy spectrum is as a consequence of the interplay between AB-flux, magnetic field and the quantum dot. We have found that the energy spectrum of the graphene quantum dot decreases with both magnetic and AB-flux field but the effect of AB-flux field is more dominant. By ameliorating the intensity of the AB-flux field and keeping the magnetic field constant, the quantum-dot states integrate to produce Landau Levels. We also found that the energy gap would be closed with increasing AB-flus field if we shift the degeneracy that appear in the energy levels via the magnetic field. The reversal symmetric are broken by the magnetic ordering.

The mathematical method utilized in this paper is very efficient and easy to use. We hope that our theoretical work will influence experimental efforts on the effects of AB-flux field and magnetic barriers on Dirac fermions.

## Declarations

### Author contribution statement

Fernando Adan Serrano Orozco, Jose Luis Cuevas Figueroa, Hugo Moises Martinez Carrada: Conceived and designed the analysis; Wrote the paper.

Juan Gerardo Avalos Ochoa, Xochitl Cabrera Rivas: Analyzed and interpreted the data; Contributed analysis tools or data; Wrote the paper.

### Funding statement

This research did not receive any specific grant from funding agencies in the public, commercial, or not-for-profit sectors.

### Competing interest statement

The authors declare no conflict of interest.

### Additional information

No additional information is available for this paper.
